# Supplementary material for: Large CRISPR-Cas-induced deletions in the oxamniquine resistance locus of the human parasite Schistosoma mansoni
Source: Wellcome Open Res. 2021 Jan 20;5:178. Originally published 2020 Jul 23. [Version 2] doi: 10.12688/wellcomeopenres.16031.2 (PMC7405262; doi:10.12688/wellcomeopenres.16031.2)
Supplement: Peer review history

# Peer review: Reviewer response for version 1

**DOI:** 10.21956/wellcomeopenres.17586.r39712

**Published:** 2020-09-01

**Authors:** Patrick Skelly

**Affiliations:** 1 Molecular Helminthology Laboratory, Department of Infectious Disease and Global Health, Cummings School of Veterinary Medicine, Tufts University, North Grafton, MA, USA

This study by Sankaranarayanan & Coghlan *et al.* employed CRISPR-Cas9 to target for disruption the SULT-OR sulfotransferase gene in *Schistosoma mansoni* using electroporation to deliver a ribonucleoprotein (RNP) complex consisting of gRNA, tracrRNA and Cas9 protein. Mutations induced by this treatment were subsequently detected by high-throughput sequencing. The results provide independent confirmation that CRISPR/Cas delivery to *S. mansoni* can indeed induce mutations in these parasites, albeit (so far) at a low level. Three developmental stages were examined, and mutations were most commonly detected in adult worms (0.3-2.0% of aligned sequence reads), followed by sporocysts (0.1-0.2%), and were extremely rare in eggs. The work provides hope that ongoing research will permit researchers to use refined methods to target the germ line to create stable knock-out or knock-in strains of any schistosome gene of interest.

 Some comments and questions:

Animal Procedures. Why were snails “moved into dark cupboards at 28°C when they start shedding cercariae” and is there a reference showing that this is helpful? Page 4: How was the water “conditioned”? (At least in my version) the “Welfare assessments….” sentence needs correcting.

Parasite Material. Useful, detailed methods are reported. So, for consistency, give specifics of the Percoll-sucrose solution.

CRISPR-Cas9 ribonucleoprotein complex assembly. Since we expect the gRNA and the nuclease to associate in a 1:1 ratio, why not mix these in equimolar amounts? How can the authors confirm that the gRNA and nuclease actually formed the RNP complex? Is there a way to determine how efficiently this occurred? Low efficiency might provide some explanation for the relatively low level of mutation detected here?

Bioinformatic analysis. Consider adding a reference to Phred Quality Scoring so that interested readers can make sense of the <23 score cut off. It may also be useful to define here “SNP” and “NHEJ”.

Results. CRISPR-Cas9 machinery…..The authors should report if ALL of the adults (males and females) yielded a similar staining pattern. In Figure 2A and Movie 1, surface staining is not uniform along the length of the worm; did all males exhibit such staining in a similar region and to a similar extent (and in all replicates)? Does any of the RNP staining co-localize with DAPI-stained nuclei? If yes, point examples out. If not, how well is the nuclear localization signal working?

Discussion. It is reported that “no evidence for CRISPR-Cas9-induced insertions or substitutions in the SULT-OR gene was observed.” For context, it would be useful to know how this equates (if possible, under comparable experimental conditions) with work in *Strongyloides*, *C. elegans*, and mammalian cell-lines.

 I disagree that because mutations were detected in 2% of aligned reads, this necessarily means that ~2% of cells were impacted.

 The control data shown in e.g. Figure 3A – were they derived from the mock treated worms or from worms treated with Cas9 only or from those treated with gRNA only? Or were data from all controls combined?

 Report how the tree shown in Figure S1A was generated. What does “PRJEA36577” etc. refer to in the figure? What is the value of showing anything outside of the area bounded by the red dashed line?

Is the work clearly and accurately presented and does it cite the current literature?

Yes

If applicable, is the statistical analysis and its interpretation appropriate?

Yes

Are all the source data underlying the results available to ensure full reproducibility?

Yes

Is the study design appropriate and is the work technically sound?

Yes

Are the conclusions drawn adequately supported by the results?

Yes

Are sufficient details of methods and analysis provided to allow replication by others?

Yes

Reviewer Expertise:

Molecular parasitology.

I confirm that I have read this submission and believe that I have an appropriate level of expertise to confirm that it is of an acceptable scientific standard.

# Response

**Published:** 2020-12-07

**Authors:** Gabriel Rinaldi

**Affiliations:** Parasite Genomics, Wellcome Trust Sanger Institute, UK

07 December 2020,

 Wellcome Open Research,

 On behalf of all the authors, we acknowledge and appreciate the efforts of the reviewers with the review of our manuscript. We have been able to revise along the lines of the recommendations, which has certainly contributed to improve our paper. Point-by-point responses are provided below: **Response (R)**

*30 Jul 2020 | for Version 1*

**Phillip LoVerde**, Department of Biochemistry and Structural Biology, University of Texas Health Science Center at San Antonio, San Antonio, TX, USA

 Schistosomiasis remains a serious global health problem. The toolbox available to scientists continues to improve. The ability to manipulate genes is a key technique to the continued success of the field to address questions of biology, host-parasite interactions, vaccines, drugs and diagnostics among others. The manuscript by the Wellcome Sanger Group is a welcome addition as it provides very detailed methods, state of the art approach and detailed analysis of the results. In fact, it provides a roadmap for others to follow. This is in spite of the fact they were not successful in producing a drug resistant transgenic strain. Clearly part of the reason was that only somatic cells were affected by the CRISPR-Cas 9 construct. They recognize that affecting germ line cells will likely make a difference. Another plausible explanation is that the cells in which CRISPR-Cas9 did induce mutations represented a small fraction of the cells expressing SmSULT-OR. They also acknowledge that the resistance trait is double recessive inferring that both alleles must be interrupted. I may have missed it but did not see where they attempted to demonstrate that both alleles of SmSULT-OR were interrupted.

**R.** We acknowledge the reviewer for the positive comments, and as indicated, the resistance trait is double recessive inferring that both alleles must be interrupted in the germline to generate a transgenic line with a fully OXA-resistant phenotype. In this study, we did not demonstrate that both alleles of SmSULT-OR were interrupted, given that the sequence data were generated from a large number of cells, probably including a mixture of wild type and mutant cells. In order to identify allele-specific mutations single cell DNA PCR and/ or sequencing approaches need to be applied (PMID: 31827197; PMID: 30992375). This can be optimised in the future, in particular after having shown that single cell sequencing approaches are feasible in schistosomes (PMID: 32973030; bioRxiv 754713; doi: https://doi.org/10.1101/754713)

 The manuscript is well-written and follows a logical progression with well-designed experiments. Suggest use the term intramolluscan instead of intrasnail.

**R. **Edited as recommended

 An issue out of their control is the difficulty in navigating the Extended data Figures and Tables. However, all the data is available to the reader.

**R.** We agree and have also found difficulties in navigating the Extended Data files. We will raise a comment to the editorial team in this regard.

*04 Aug 2020 | for Version 1*

**Arnon Jurberg**, Oswaldo Cruz Foundation, Rio de Janeiro, Brazil

 The work by Sankaranarayanan & Coghlan et al. sought to deploy CRISPR/Cas9 by electroporation at distinct developmental stages of schistosomes (more specifically, eggs, sporocysts and adult worms). A successful implementation of this technique in schistosomes will open up numerous possibilities for the study of gene function in these flatworms and is likely to revolutionize the field in the same manner as gene knockout by homologous recombination in mice and rats.

 The paper is well written and provided a detailed description of their methods and findings. The figures are well conceived and the paper is likely to provide grounds for others to come, although CRISPR efficiency rate was below 5%.

 1. Considering the low editing efficiency, did the authors evaluate the activity of other gRNAs against SULT-OR?

**R. **This is a good question, and indeed we have originally designed three gRNAs in total, but have only tested one of them so far. The gRNA employed in this study is the closest to the start codon of the exon 1 (Figure 1B), and predicted to be SmSULT-OR specific; therefore, reducing the chances of off-target mutations in the other members of the sulfotransferase family (as shown in Figure S3 A). Additionally, it is expected that an indel mutation (that is not a multiple of 3 bp in size) at the 5' end of the gene could disrupt the reading frame of nearly the whole gene and so nearly the whole protein, while such a mutation near the 3' end of the gene would only affect the sequence of the end of the protein.  

 2. It also caught my attention the apparent lack of nuclear staining for fluorescently labelled Cas9-gRNA. Did the authors address this?

**R. **We agree with the reviewer that nuclear staining, i.e. co-staining of the nuclei with DAPI and ATTO ^TM^ 550 is not evident. Probably the confocal imaging is not sensitive enough to detect the fluorescent ribonucleoprotein complex within the cell nucleus. However, the Cas9 nuclease contains a nuclear localization sequence, and we identified site-specific deletions across the expected double-strand break site in the three developmental stages tested herein. This strongly suggests that even though no nuclear signal was detected (as above, probably due to sensitivity of the confocal assay), the ribonucleoprotein complex did indeed reach the genomic DNA of the cells. In this regard, we have now incorporated the following statement in Discussion (4 ^th^ paragraph):” *The absence of nuclear localisation of the RNP complex in the confocal images may indicate that the 4-hour timeframe between the molecule delivery and parasite fixation was too short, and which may explain, at least partially, the low CRISPR-induced deletion efficiency.”*

 3. It was also unclear to me whether the staining pattern between adult males and females were similar, and I would like to suggest the addition of further details. An alternative approach that can assist in achieving greater CRISPR efficiencies in schistosomes is the use of microinjection (perhaps in the ovary of females).

**R. **No evident differences were observed in the staining pattern between adult males and females. Accordingly, we have incorporated the following sentence in the Results (section “CRISPR-Cas9 machinery successfully delivered into schistosome developmental stages”): *“...no evident differences in the staining pattern were evident between sexes”. *Regarding the use of microinjection to introduce the CRISPR machinery into the germ line, it is a very interesting idea worth testing in future experiments.

 4. Once mutations in the target gene are predicted to induce oxamniquine resistance, did the authors evaluate whether incubation of electroporated parasites with this drug could improve CRISPR efficiency?

**R. **We acknowledge this suggestion, and indeed in one experiment we incubated CRISPR-mutated worms in the presence of oxamniquine (OXA), but no obvious resistant phenotype was detected. These findings were not unexpected, given the intrinsic low efficiency of CRISPR and that only a few (presumably somatic) cells, were mutated in the whole parasite. In the current study, we decided to focus on describing the induced deletions as a *proof-of-principle* that the technology can be applied to several developmental stages, in this case as a “somatic transgenesis” approach. Further experiments will be performed in the future to investigate the development of an OXA-resistance phenotype.

*24 Aug 2020 | for Version 1*

**Mattie Pawlowic**, Biological Chemistry and Drug Discovery, Wellcome Centre for Anti-Infectives Research, University of Dundee, Dundee, UK

 Sankaranarayanan and colleagues report their work to mutate *Schitosoma mansoni* SULT-OR, a gene related to oxamniquine resistance, using transfection of ribonucleoprotein (RNP) complex to induce CRISPR/Cas9 double-stranded breaks. They use this approach on worm adults, sporocysts, and eggs, and deep sequence the resulting parasites to determine which mutations are present. This represents a new approach for genetic modification of Schistosoma.

 The authors find that adult and sporocysts (not eggs) take up fluorescently labelled RNPs. These bind non-specifically on the surface of adult worms and also concentrate in the gut. In the future, the authors should visualise what uptake may occur in adult worms without electroporation, as it appears a significant amount of uptake may be due to feeding. It is curious that the Cas9, which contains a nuclear localisation sequence, does not localise the RNP complex to the nucleus of worm cells. It could be that the 4-hour time frame is too short to observe this, or it could be that lack of nuclear localisation may explain the low editing efficiency.

**R.** Although the fluorescence signal is faint in eggs, in particular compared to adult worms and sporocysts, Figures 2F and H show it is inside the eggshell and in the miracidium surface. Given the high concentration of RNP in cells lining the gut, as suggested by the reviewer, in future experiments we may compare the RNP delivery by soaking and electroporation. Regarding the absence of co-localisation of DAPI and ATTO ^TM^ 550, please see the answer to question 2 of the previous reviewer. The 4-hour timeframe being too short is a good hypothesis and worth testing it in future experiments. In this regard, we added the following statement in the 4th paragraph of Discussion: *“Future experiments comparing the delivery of the RNP complex by soaking versus electroporation would inform about the best delivery approach. The absence of nuclear localisation of the RNP complex in the confocal images may indicate that the 4-hour timeframe between the molecule delivery and parasite fixation was too short, and which may explain, at least partially, the low CRISPR-induced deletion efficiency.”*

 After 4 days of culture, the authors extracted DNA from transfected parasites, PCR amplified the region of interest, and deep sequenced to identify mutations. I understand that a larger PCR fragment is required to identify deletions, however the authors designed the ends of the PCR products to overlap at the Cas9 cut site. This led to difficulties in identifying true mutations vs sequencing errors at the cut site. Therefore, I think indels may be under-reported as they were not well captured.

**R.** In Figure 1C, we show the positions of the PCR primers in green and the amplicon in pale blue. The amplicon length is 262bp and it covers the predicted Cas9 site (position 137), the PCR products do not overlap at the Cas9 cut site. On the other hand, the forward and reverse sequence reads do overlap the cut site, increasing the accuracy to detect CRISPR-induced mutations. To make the Figure clearer, we have now incorporated more information in the Figure 1C legend as follows: *“(C) Reference PCR amplicon (pale blue), showing the positions of the gRNA, PAM, DSB, forward and reverse PCR primers (green), and forward and reverse sequence reads (orange), as well as a SNP site found in many sequence reads. The diagrams are drawn to scale.”*

 The authors find that deletions are the most common mutations the occur at the SULT-OR locus. I disagree that deletions in the range of 24-102 bp should be called "large". Consistent with use of CRISPR in other systems, deletions occur upstream of the cut site. Although adult worms took up RNPs the best, the mutation rate was found to be only <2%. This is likely due to the RNPs not transfecting every cell in the adult worm.

**R**. As such, the term “large” is relative, and we agree that deletions in the range of 24-102 are not large compared to deletions of hundreds and even thousands of bases. However, given that in the earlier CRISPR studies the majority of identified deletions were in the range of 1-3bp around the double-stranded break site (see https://www.biorxiv.org/content/10.1101/2020.08.05.216739v1), and our study is the second in schistosomes, and the first one in this parasite showing deletions up to 102bp, we have decided to keep the term “large” to describe them. Finally, even longer deletions (~ 500bp) as shown for *Strongyloides* spp (PMID: 29016680) but not detectable by our PCR-based approach, cannot be ruled out in our system.

 Despite SULT-OR being at its highest expression levels in adult worms, mutation rates were too low to see an impact on SULT-OR expression levels.

 Overall this work reports new methods for genetic modification of *Schistosoma mansoni*. While the efficiency is low, further optimisation makes this approach promising.

**R.** We completely agree with the reviewer, and further optimisation, not only to increase the CRISPR-induced mutations, but also to introduce them into the germline, is planned. 

*01 Sep 2020 | for Version 1*

**Patrick Skelly**, Molecular Helminthology Laboratory, Department of Infectious Disease and Global Health, Cummings School of Veterinary Medicine, Tufts University, North Grafton, MA, USA

 This study by Sankaranarayanan & Coghlan *et al.* employed CRISPR-Cas9 to target for disruption the SULT-OR sulfotransferase gene in *Schistosoma mansoni* using electroporation to deliver a ribonucleoprotein (RNP) complex consisting of gRNA, tracrRNA and Cas9 protein. Mutations induced by this treatment were subsequently detected by high-throughput sequencing. The results provide independent confirmation that CRISPR/Cas delivery to *S. mansoni* can indeed induce mutations in these parasites, albeit (so far) at a low level. Three developmental stages were examined, and mutations were most commonly detected in adult worms (0.3-2.0% of aligned sequence reads), followed by sporocysts (0.1-0.2%), and were extremely rare in eggs. The work provides hope that ongoing research will permit researchers to use refined methods to target the germ line to create stable knock-out or knock-in strains of any schistosome gene of interest.

 Some comments and questions:

Animal Procedures. Why were snails “moved into dark cupboards at 28°C when they start shedding cercariae” and is there a reference showing that this is helpful? Page 4: How was the water “conditioned”? (At least in my version) the “Welfare assessments….” sentence needs correcting.

**R. **Regarding the question “why the snails were moved into dark cupboards at 28°C when they start shedding cercariae”, there is not a reference showing this is helpful to obtain more cercariae. In order to experimentally obtain the highest number of cercariae, as far as we are aware, there are mainly two approaches used by most of the laboratories working with schistosomes. One approach involves keeping the patent snails under 12-hour light/dark cycles and 24 hours before shedding the snails are moved to the dark as described (PMID: 24510597). A second approach, that originally was followed in David Dunne laboratory at the Department of Pathology, University of Cambridge (personal communication by Anna Protasio), and that we inherited involves moving the snails into dark incubators when they start to shed cercariae, and keeping them at dark all the time. Even though, as far as we are aware, no studies comparing the two approaches have been conducted, the number of cercariae obtained by the latter approach, currently used by us, has been high enough to carry out all the experiments described in the current manuscript, in addition to maintaining the whole life cycle independently at the Wellcome Sanger Institute and supporting the rest of the schistosoma-related projects.  

**R. **In the Methods section “Animal Procedures” we have now included the reference for the conditioned water recipe (i.e. PMID: 24510597)

**R. **We have now edited the sentence ‘ *Welfare assessments are carried out daily abnormal signs of behaviour or clinical signs of concern are reported*.’ as follows: ‘ *Welfare assessments are carried out daily, and abnormal signs of behaviour or clinical signs of concern are reported.*’

Parasite Material. Useful, detailed methods are reported. So, for consistency, give specifics of the Percoll-sucrose solution.

**R. **Following the reviewer’s suggestion we have now included more details about the Percoll-sucrose solution (in Methods, “Parasite Material”), as follows: *‘The filtrate was passed through a Percoll-sucrose gradient prepared by mixing 8 ml of Percoll with 32 ml of sterile-filtered 0.25M sucrose…’* ** 

CRISPR-Cas9 ribonucleoprotein complex assembly. Since we expect the gRNA and the nuclease to associate in a 1:1 ratio, why not mix these in equimolar amounts? How can the authors confirm that the gRNA and nuclease actually formed the RNP complex? Is there a way to determine how efficiently this occurred? Low efficiency might provide some explanation for the relatively low level of mutation detected here?

**R.** Indeed, as the reviewer pointed out we did not use equimolar amounts of gRNA and nuclease. For the RNP complex assembly we followed the protocol suggested by the manufacturer IDT, slightly modified based on a well-optimised protocol to introduce CRISPR-Cas9 mutations by RNP complex in pluripotent stem cells (PMID: 30912046). In these protocols an excess of gRNA compared to Cas9 nuclease is employed, presumably to ensure that all the nuclease molecules are combined with the gRNA. Having said that, future experiments could be conducted to test the CRISPR efficiency by changing the relative concentrations of the RNP complex components. We have now provided in the manuscript further information about the RNP assembly protocol. In the Methods (section, “CRISPR-Cas 9 ribonucleoprotein complex assembly”) we have included the following statement: ‘ *The CRISPR-Cas9 ribonucleoprotein complex (RNP) was assembled in vitro following the manufacturer recommendations slightly modified based on* *(Bruntraeger et al. 2019)* *by combining…’*

**R.** We have not confirmed the actual RNP complex was properly assembled, but the presence of definitive CRISPR-Cas -induced deletions around the predicted double-stranded break site strongly suggests that the complex was, at least partially, active. Protocols to evaluate the assembly of Cas9/gRNA complexes by using a fluorometric molecular beacon-like assay have been developed (PMID: 26945042), and could be applied in our future studies. We agree with the reviewer that low efficiency of assembly of RNP complexes could, at least partially, explain the low efficiency of the system to induce site-specific mutations. However, the CRISPR efficiency reported by Ittiprasert et al (PMID: 30644357), where RNP complex and lentivirus were (separately) employed to induce site-specific mutations, was even lower than that reported here. This suggests that the low efficiency may be related to the species rather than the approach employed to deliver the CRISPR-Cas cargo.  

**R. **In the first paragraph of Discussion we have now included the following sentence to address this point raised by the reviewer: *‘The low CRISPR-Cas efficiency in our study may be explained, at least partially, by low efficiency of RNP complex assembly; however, as discussed above, a low CRISPR-Cas efficiency was also described when lentivirus (rather than RNP complex) was used to deliver the CRISPR-Cas9 cargo into the parasite* *(Ittiprasert et al. 2019)* *’.*

Bioinformatic analysis. Consider adding a reference to Phred Quality Scoring so that interested readers can make sense of the <23 score cut off. It may also be useful to define here “SNP” and “NHEJ”.

**R.** Edited as suggested.

Results. CRISPR-Cas9 machinery…..The authors should report if ALL of the adults (males and females) yielded a similar staining pattern. In Figure 2A and Movie 1, surface staining is not uniform along the length of the worm; did all males exhibit such staining in a similar region and to a similar extent (and in all replicates)? Does any of the RNP staining co-localize with DAPI-stained nuclei? If yes, point examples out. If not, how well is the nuclear localization signal working?

**R.** Following the reviewer’s suggestion we have now included in Results (section

 “CRISPR-Cas9 machinery successfully delivered into schistosome developmental stages”) the following statement: *‘The staining pattern was similar in all observed specimens, and no evident differences in the staining pattern were evident between sexes* *.’ *Regarding the absence of co-localisation of DAPI and ATTO ^TM^ 550, please see the answer to question 2 of the previous reviewer (Arnon Jurberg). We have not evaluated how well the nuclear localisation signal worked in our system. However, the presence of definitive CRISPR-Cas -induced deletions around the predicted double-stranded breaking points strongly suggests the system worked.

Discussion. It is reported that “no evidence for CRISPR-Cas9-induced insertions or substitutions in the SULT-OR gene was observed.” For context, it would be useful to know how this equates (if possible, under comparable experimental conditions) with work in *Strongyloides*, *C. elegans*, and mammalian cell-lines.

**R.** Following the reviewer’s suggestion we have now included in the first paragraph of Discussion the following statement: ‘ *In Strongyloides spp, Gang et al (Gang et al. 2017) showed that in the absence of a repair template, small insertions or deletions (indels) or substitutions were not observed, but instead the authors found deletions of >500 bp at the target site in the unc-22 gene. On the other hand, in C. elegans, one study in the absence of a repair template, detected deletions ranging from 7 bp to >2 kb in the dpy-11 and unc-4 genes (Chiu et al. 2013); while another study found only small insertions and deletions of <20 bp (Friedland et al. 2013)’. In mammalian cell lines, the majority of indels are relatively small (1-50 bp), but larger deletions of kilobases in size are also sometimes observed (Kosicki et al, https://www.biorxiv.org/content/10.1101/2020.08.05.216739v1).’*

 I disagree that because mutations were detected in 2% of aligned reads, this necessarily means that ~2% of cells were impacted.

**R. **We agree that in order to claim that 2% of aligned reads means that ~2% of cells were impacted, we have to assume an even distribution of mutations, and this is not necessarily the case. In Discussion (4 ^th^ paragraph) we have now edited the text accordingly: *‘The large deletions spanning the predicted Cas9 cut site were found in 0.3–2.0% of aligned reads from CRISPR-treated adult worms, so our best estimate of the fraction of adult cells in which CRISPR worked is 0.3–2.0% assuming an even distribution of mutations across the transfected parasites. However, since a pool of five adult worms were transfected with the RNP complex, the efficiency of CRISPR (and the amount of knock-down at the mRNA level) may have varied between worms, as well as between cells of an individual worm.’*

 The control data shown in e.g. Figure 3A – were they derived from the mock treated worms or from worms treated with Cas9 only or from those treated with gRNA only? Or were data from all controls combined?

**R.** For both experimental samples and controls in all the figures we provide the sample identifiers. The information for all these identifiers and samples can be found in Supplementary Table 1. In particular for Figure 3A the controls include worm-only controls for the adult samples, worm+Cas9-only and worm-only controls for the sporocyst samples, egg+Cas9-only and egg-only controls for the egg samples. To clarify this point, we have now added this information in the Figure 3A legend as follows: *‘(A) Frequency of deletions in NGS sequencing data, identified with the assistance of CRISPResso in three biological replicates from adults, two from sporocysts, and three from eggs, as indicated (sample identifiers at the bottom). The controls include worm-only controls for the adult samples, worm treated with Cas9-only and worm-only controls for the sporocyst samples, eggs treated with Cas9-only and egg-only controls for the egg samples.’*

 Report how the tree shown in Figure S1A was generated. What does “PRJEA36577” etc. refer to in the figure? What is the value of showing anything outside of the area bounded by the red dashed line?

**R. **To clarify this point we have now included two sentences in the figure legend describing how the tree shown in Figure S1A (now in Extended data V2) was generated and the meaning of identifiers next to the species names, as follows: *‘The phylogenetic tree was generated by WormBase ParaSite using the EnsemblCompara pipeline [34]. Identifiers beside the species names in the tree indicate the NCBI BioProject accession numbers for the sequencing projects for each species or species strain.’*

**R.** The red dashed line shows a clade of the phylogenetic tree that includes paralogues of SULT-OR that lie in a region about 3.2 Mb along *S. mansoni* chromosome 6. The rest of the phylogenetic tree, outside the red dashed line, includes other more distant paralogues of SULT-OR, that are scattered around the *S. mansoni *genome. We think it could be of interest to readers that, as well as several closely related paralogues of SULT-OR that are found nearby to SULT-OR in the genome, the SULT-OR gene also has more distantly related paralogues scattered around the genome (these are the ones outside the red dashed line).

# Peer review: Reviewer response for version 1

**DOI:** 10.21956/wellcomeopenres.17586.r39705

**Published:** 2020-08-24

**Authors:** Mattie Pawlowic

**Affiliations:** 1 Biological Chemistry and Drug Discovery, Wellcome Centre for Anti-Infectives Research, University of Dundee, Dundee, UK

Sankaranarayanan and colleagues report their work to mutate *Schitosoma mansoni* SULT-OR, a gene related to oxamniquine resistance, using transfection of ribonucleoprotein (RNP) complex to induce CRISPR/Cas9 double-stranded breaks. They use this approach on worm adults, sporocysts, and eggs, and deep sequence the resulting parasites to determine which mutations are present. This represents a new approach for genetic modification of Schistosoma. 

 The authors find that adult and sporocysts (not eggs) take up fluorescently labelled RNPs. These bind non-specifically on the surface of adult worms and also concentrate in the gut. In the future, the authors should visualise what uptake may occur in adult worms without electroporation, as it appears a significant amount of uptake may be due to feeding. It is curious that the Cas9, which contains a nuclear localisation sequence, does not localise the RNP complex to the nucleus of worm cells. It could be that the 4-hour time frame is too short to observe this, or it could be that lack of nuclear localisation may explain the low editing efficiency.

 After 4 days of culture, the authors extracted DNA from transfected parasites, PCR amplified the region of interest, and deep sequenced to identify mutations. I understand that a larger PCR fragment is required to identify deletions, however the authors designed the ends of the PCR products to overlap at the Cas9 cut site. This led to difficulties in identifying true mutations vs sequencing errors at the cut site. Therefore, I think indels may be under-reported as they were not well captured.

 The authors find that deletions are the most common mutations the occur at the SULT-OR locus. I disagree that deletions in the range of 24-102 bp should be called "large". Consistent with use of CRISPR in other systems, deletions occur upstream of the cut site. Although adult worms took up RNPs the best, the mutation rate was found to be only <2%. This is likely due to the RNPs not transfecting every cell in the adult worm. 

 Despite SULT-OR being at its highest expression levels in adult worms, mutation rates were too low to see a impact on SULT-OR expression levels.

 Overall this work reports new methods for genetic modification of *Schistosoma mansoni*. While the efficiency is low, further optimisation makes this approach promising.

Is the work clearly and accurately presented and does it cite the current literature?

Yes

If applicable, is the statistical analysis and its interpretation appropriate?

Yes

Are all the source data underlying the results available to ensure full reproducibility?

Yes

Is the study design appropriate and is the work technically sound?

Yes

Are the conclusions drawn adequately supported by the results?

Yes

Are sufficient details of methods and analysis provided to allow replication by others?

Yes

Reviewer Expertise:

Parasite genetics and biochemistry, Cryptosporidium.

I confirm that I have read this submission and believe that I have an appropriate level of expertise to confirm that it is of an acceptable scientific standard.

# Peer review: Reviewer response for version 1

**DOI:** 10.21956/wellcomeopenres.17586.r39711

**Published:** 2020-08-04

**Authors:** Arnon Jurberg

**Affiliations:** 1 Oswaldo Cruz Foundation, Rio de Janeiro, Brazil

The work by Sankaranarayanan & Coghlan *et al.* sought to deploy CRISPR/Cas9 by electroporation at distinct developmental stages of schistosomes (more specifically, eggs, sporocysts and adult worms). A successful implementation of this technique in schistosomes will open up numerous possibilities for the study of gene function in these flatworms and is likely to revolutionize the field in the same manner as gene knockout by homologous recombination in mice and rats.

 The paper is well written and provided a detailed description of their methods and findings. The figures are well conceived and the paper is likely to provide grounds for others to come, although CRISPR efficiency rate was below 5%.

1. Considering the low editing efficiency, did the authors evaluate the activity of other gRNAs against *SULT-OR*?
2. It also caught my attention the apparent lack of nuclear staining for fluorescently labelled Cas9-gRNA. Did the authors address this?
3. It was also unclear to me whether the staining pattern between adult males and females were similar, and I would like to suggest the addition of further details. An alternative approach that can assist in achieving greater CRISPR efficiencies in schistosomes is the use of microinjection (perhaps in the ovary of females).
4. Once mutations in the target gene are predicted to induce oxamniquine resistance, did the authors evaluate whether incubation of electroporated parasites with this drug could improve CRISPR efficiency?

Is the work clearly and accurately presented and does it cite the current literature?

Yes

If applicable, is the statistical analysis and its interpretation appropriate?

Yes

Are all the source data underlying the results available to ensure full reproducibility?

Yes

Is the study design appropriate and is the work technically sound?

Yes

Are the conclusions drawn adequately supported by the results?

Yes

Are sufficient details of methods and analysis provided to allow replication by others?

Yes

Reviewer Expertise:

Developmental biology, molecular biology and gene editing, cell biology.

I confirm that I have read this submission and believe that I have an appropriate level of expertise to confirm that it is of an acceptable scientific standard.

# Peer review: Reviewer response for version 1

**DOI:** 10.21956/wellcomeopenres.17586.r39710

**Published:** 2020-07-30

**Authors:** Phillip LoVerde

**Affiliations:** 1 Department of Biochemistry and Structural Biology, University of Texas Health Science Center at San Antonio, San Antonio, TX, USA

Schistosomiasis remains a serious global health problem. The toolbox available to scientists continues to improve. The ability to manipulate genes is a key technique to the continued success of the field to address questions of biology, host-parasite interactions, vaccines, drugs and diagnostics among others. The manuscript by the Wellcome Sanger Group is a welcome addition as it provides very detailed methods, state of the art approach and detailed analysis of the results. In fact, it provides a roadmap for others to follow. This is in spite of the fact they were not successful in producing a drug resistant transgenic strain. Clearly part of the reason was that only somatic cells were affected by the CRISPR-Cas 9 construct. They recognize that affecting germ line cells will likely make a difference. Another plausible explanation is that the cells in which CRISPR-Cas9 did induce mutations represented a small fraction of the cells expressing SmSULT-OR. They also acknowledge that the resistance trait is double recessive inferring that both alleles must be interrupted. I may have missed it but did not see where they attempted to demonstrate that both alleles of SmSULT-OR were interrupted.

 The manuscript is well-written and follows a logical progression with well-designed experiments.

 Suggest use the term intramolluscan instead of intrasnail.

 An issue out of their control is the difficulty in navigating the Extended data Figures and Tables. However, all the data is available to the reader.

Is the work clearly and accurately presented and does it cite the current literature?

Yes

If applicable, is the statistical analysis and its interpretation appropriate?

Yes

Are all the source data underlying the results available to ensure full reproducibility?

Yes

Is the study design appropriate and is the work technically sound?

Yes

Are the conclusions drawn adequately supported by the results?

Yes

Are sufficient details of methods and analysis provided to allow replication by others?

Yes

Reviewer Expertise:

Schistosomiasis host-parasite interactions involving molecular, immunological and genetic approaches. Drug Discovery.

I confirm that I have read this submission and believe that I have an appropriate level of expertise to confirm that it is of an acceptable scientific standard.
